# Editorial: Update on Multidisciplinary Management of Surgical Neurovascular Pathologies

**DOI:** 10.3389/fsurg.2022.923493

**Published:** 2022-06-07

**Authors:** Sabino Luzzi, Alfio Spina, Alice Giotta Lucifero

**Affiliations:** ^1^ Neurosurgery Unit, Department of Clinical-Surgical, Diagnostic and Pediatric Sciences, University of Pavia, Pavia, Italy; ^2^ Neurosurgery Unit, Department of Surgical Sciences, Fondazione IRCCS Policlinico San Matteo, Pavia, Italy; ^3^ Department of Neurosurgery and Gamma Knife Radiosurgery, IRCCS San Raffaele Scientific Institute, Vita-Salute San Raffaele University, Milan, Italy

**Keywords:** neurosurgery, surgical neurovascular pathologies, neurovascular, surgical procedures, arteriovenous malfomations

**Editorial on the Research Topic**

***Update on Multidisciplinary Management of Surgical Neurovascular Pathologies***

Neurovascular pathology represents one of the most important problems of neurosurgical practice. With the advancement of knowledge and technology, the updating of skills and treatment strategies for these very different pathologies is of fundamental importance. This Special Issue aims to provide the elements of a more updated vision of the pathology, while also considering multidisciplinary aspects that cannot be ignored.

We want to warmly thank all the Authors who contributed to the Research Topic “Update on Multidisciplinary Management of Surgical Neurovascular Pathologies” with their valuable articles.

In the original research “*Optic Foraminotomy for Clipping of Superior Carotid-Ophthalmic Aneurysms*” (1), Dr. Baldoncini et al. stressed the advantages of the optic foraminotomy for clip ligation of carotid-ophthalmic aneurysms. Dr. Chen and co-workers highlighted the advantages of performing direct carotid artery exposure for acute cerebral infarction in a hybrid angiography suite in their article titled “*Direct Carotid Artery Exposure for Acute Cerebral Infarction in Hybrid Angiography Suite: Indications and Limitations*” (2). Dr. Tanabe and colleagues, in the original article “*Staged Hybrid Techniques with Straightforward Bypass Surgery Followed by Flow Diverter Deployment for Complex Recurrent Middle Cerebral Artery Aneurysm*” (3), described their hybrid technique consisting of flow replacement bypass and flow diverter deployment for complex recurrent middle cerebral artery aneurysms. In their article “*Delayed Progressive Mass Effect After Secured Ruptured Middle Cerebral Artery Aneurysm: Risk Factors and Outcomes*” (4), Dr. Li and coworkers explored the risk and predictive factors of poor outcomes for those patients with a progressive mass effect after endovascular or surgical treatment of ruptured MCA aneurysms and underwent salvage surgery. In the article “*The different fates of three aneurysms: Diagnosis and treatment strategies for unruptured intracranial aneurysms with other intracranial diseases*” (5), Dr. Gaochao Guo and co-workers analyzed the possible treatment strategies for intracranial aneurysms complicated by other diseases in three different clinical scenarios. Lastly, in their interesting manuscript “*Monocyte count on admission is predictive of shunt-dependent hydrocephalus after aneurysmal subarachnoid hemorrhage*” (6), Dr. Cuoco and colleagues interestingly found that monocyte count ≥0.80 × 103/uL at admission predicts shunt-dependent hydrocephalus in patients with aneurysmal subarachnoid hemorrhage.

All these articles contributed to furtherly expanding the volume of knowledge about the complex field of neurovascular pathology, this point is the main target of this Special Issue.

We appreciated the rigorous methodology and brilliant discussions of each study. Moreover, we appreciated the effort of the Reviewers who supported the peer review process. Proud of the commendable work done by all the eminent Contributors, we want to thank them once again, along with Frontiers of Neurosurgery, for the great opportunity to have edited this Special Issue.
